# Chronic bronchiectasis and celiac disease: An uncommon association and the importance of a comprehensive diagnostic approach

**DOI:** 10.1002/ccr3.9550

**Published:** 2024-11-03

**Authors:** Paul Ohanian, Joe Khodeir, Marielena Ohanian

**Affiliations:** ^1^ Faculty of Medicine and Medical Sciences, Department of Family Medicine, Saint Georges Hospital University Medical Center University of Balamand Beirut Lebanon; ^2^ Faculty of Medicine and Medical Sciences, Department of Dermatology, Saint Georges Hospital University Medical Center University of Balamand Beirut Lebanon; ^3^ Faculty of Medicine and Medical Sciences, Department of Geriatric Medicine, Nimes Hospital University Medical center University of Balamand Beirut Lebanon

**Keywords:** bronchiectasis, celiac disease, chronic respiratory symptoms, diagnosis and management

## Abstract

In unexplained bronchiectasis, particularly with concurrent gastrointestinal symptoms or evidence of malabsorption, consider celiac disease as a potential underlying condition. Early diagnosis and a multidisciplinary approach integrating both respiratory and gastrointestinal management can significantly enhance patient outcomes.

## 1 INTRODUCTION

Bronchiectasis is a chronic lung condition characterized by the irreversible dilation of the bronchi, leading to recurrent infections and persistent respiratory symptoms. The causes of bronchiectasis are diverse, ranging from infections and genetic conditions to immune disorders.^1^ Celiac disease, an autoimmune disorder triggered by gluten, primarily affects the gastrointestinal system but can have widespread systemic effects.^2^ Although the association between celiac disease and bronchiectasis is uncommon, it remains an important consideration in cases of unexplained bronchiectasis. This report discusses a 40‐year‐old female with undiagnosed chronic bronchiectasis and newly diagnosed celiac disease, highlighting the need for a comprehensive evaluation in such patients.

## 2 CASE HISTORY/EXAMINATION

A 40‐year‐old female patient, with no significant past medical or surgical history and a nonsmoker, presented to the emergency department with worsening dyspnea and productive cough. The patient reported that 2 weeks prior, her baseline cough, which was productive of yellow sputum, had intensified. Additionally, her baseline dyspnea had progressively worsened over the last 2 days, to the point where she experienced breathlessness even at rest.

The patient mentioned a history of chronic productive cough and exertional dyspnea for the past 5 years. However, she had not sought any medical care for these symptoms. She denied experiencing chest pain, palpitations, orthopnea, paroxysmal nocturnal dyspnea (PND), sore throat, dysphagia, dyspepsia, abdominal pain, nausea, vomiting, diarrhea, arthralgia, myalgia, dysuria, frequency, or any recent sick contacts. Notably, she reported a recent unintentional weight loss of approximately 10 kg over the past 2 years and occasional episodes of abdominal pain with frequent bloating.

On examination, the patient was tachypneic with a respiratory rate of 18 breaths per minute and an oxygen saturation of 88% on room air. Lung auscultation revealed diffuse expiratory wheezing and bilateral basilar crackles. Abdominal examination showed moderate distention with tenderness across all four quadrants.

## 3 METHODS (DIFFERENTIAL DIAGNOSIS, INVESTIGATIONS AND TREATMENT)

Arterial blood gas (ABG) analysis demonstrated hypoxia with a PaO2 of 50 mmHg and respiratory alkalosis. The patient was initiated on 5 L/min oxygen via nasal cannula. Blood, sputum cultures, and a respiratory viral panel were obtained. Laboratory investigations revealed leukocytosis (WBC count of 15,000/μL with 78% neutrophils), microcytic anemia (hemoglobin 10.5 g/dL, MCV 68 fL), and iron deficiency (low ferritin and serum iron with elevated total iron‐binding capacity [TIBC]). Inflammatory markers were elevated, with a C‐reactive protein (CRP) of 10.4 mg/L (normal range: <5 mg/L). Additionally, low levels of folic acid, vitamin B12, and vitamin D were noted, while liver function tests (LFTs) and the metabolic panel were within normal limits. Pulmonary function tests revealed a mild obstructive pattern, with an FEV1/FVC ratio of 68% and an FEV1 of 78% of the predicted value.

**FIGURE 1:**
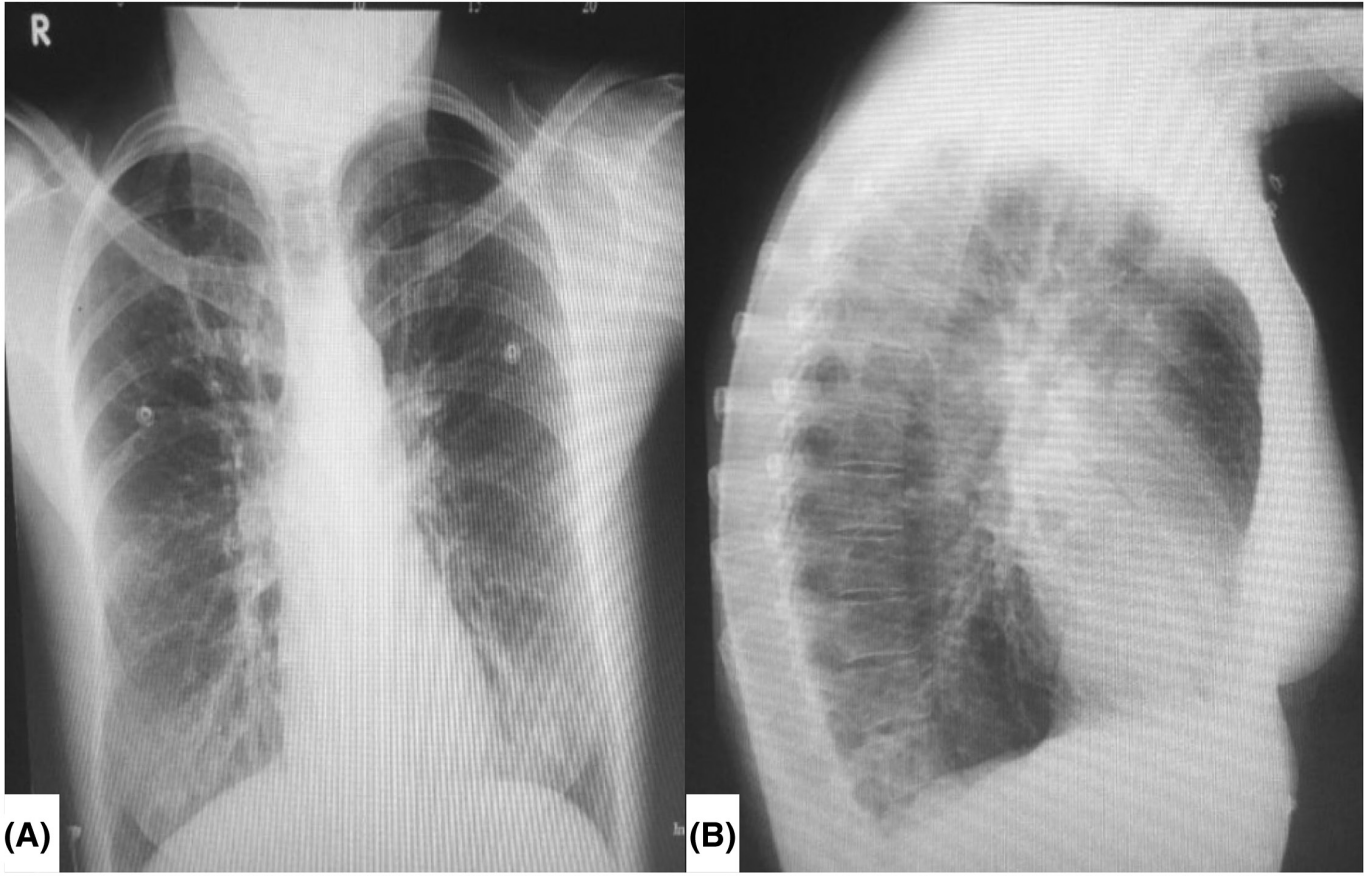
Chest x‐ray (A) AP view, and (B) lateral view, revealed hyperinflated lungs with emphysematous changes predominantly in the upper lobes and bronchiectatic changes in the lower lobes.

**FIGURE 2:**
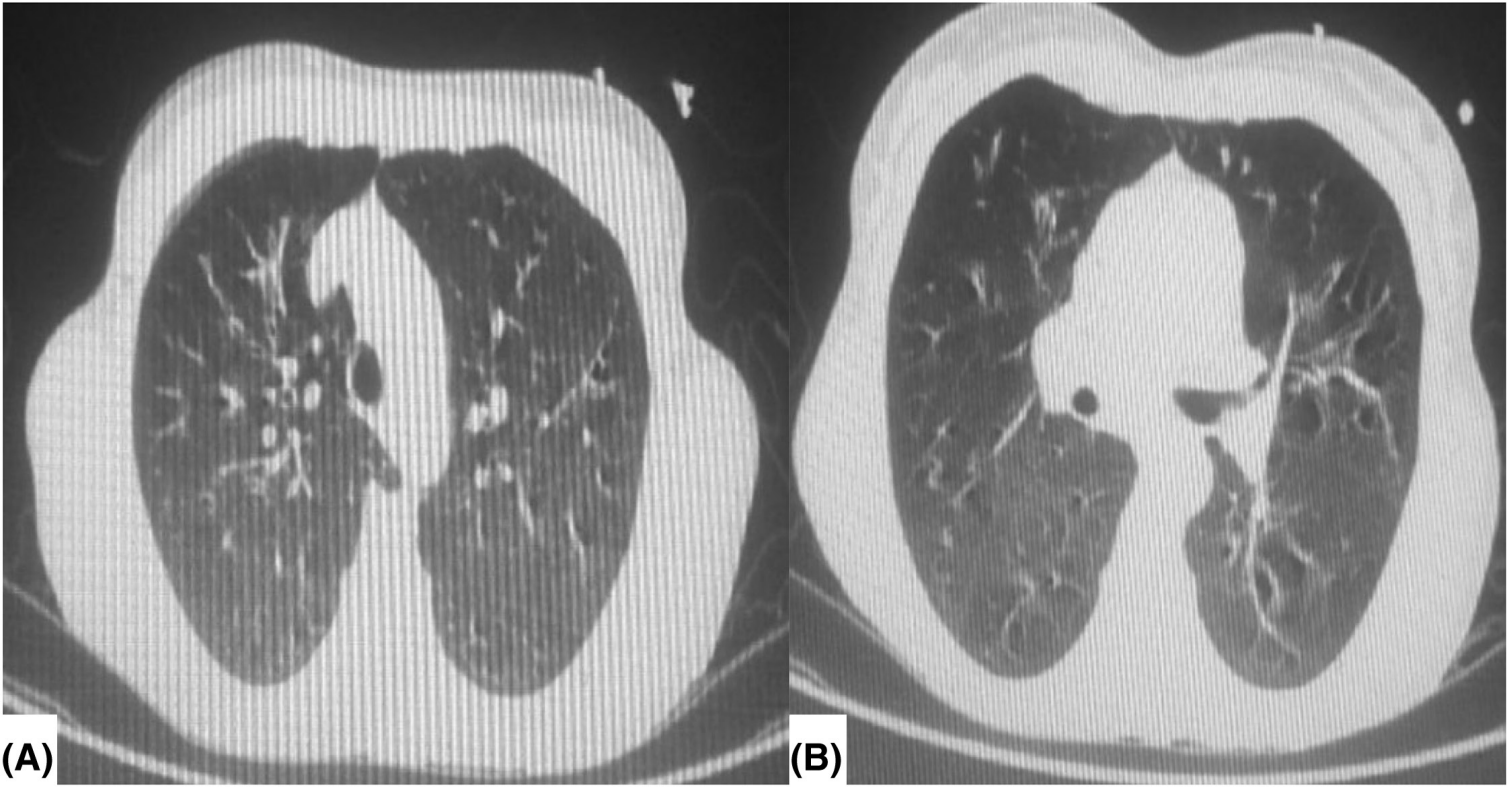
Chest CT transverse planes (A) and (B) show bilateral multifocal multilobular bronchiectasis with peribronchial thickening, consistent with acute bronchitis superimposed on chronic bronchitis.

A chest x‐ray (Figure 1A,B) revealed hyperinflated lungs with emphysematous changes predominantly in the upper lobes and bronchiectatic changes in the lower lobes, prompting further evaluation with a CT scan. The chest CT (Figure 2A,B) confirmed bilateral multifocal multilobular bronchiectasis with peribronchial thickening, consistent with acute bronchitis superimposed on chronic bronchitis. The fecal immunochemical test (FIT) was positive, and the respiratory viral panel was negative.

For the acute exacerbation, the patient was treated with inhaled salbutamol and normal saline via nebulizer, intravenous methylprednisolone (60 mg), intravenous levofloxacin (750 mg), and the mucolytic dornase alfa. Following this therapy, her dyspnea improved, and her oxygen saturation normalized.

An extensive evaluation was conducted to determine the underlying cause of her bronchiectasis. A tuberculin skin test (PPD) was negative, and chest CT did not show any signs of hilar adenopathy typical of tuberculosis. All sputum cultures, including bacterial, mycobacterial, and fungal, were negative, as was the NTM PCR from sputum. Congenital anatomical defects (tracheobronchial, vascular, and lymphatic) were ruled out based on history and imaging. Immunodeficiency was excluded with normal immunoglobulin classes and subclasses, and the patient denied any history of recurrent respiratory tract infections. Sweat chloride testing and genetic testing were negative for cystic fibrosis. Alpha‐1‐antitrypsin level was within the normal range.

A comprehensive autoimmune workup, including anti‐CCP, rheumatoid factor (RF), ANA profile, anti‐SSA, and anti‐SSB, was negative, ruling out rheumatoid arthritis and Sjögren's syndrome. Gastroscopy and colonoscopy were performed to investigate potential inflammatory bowel disease or other gastrointestinal etiologies, but both were unremarkable.

**FIGURE 3:**
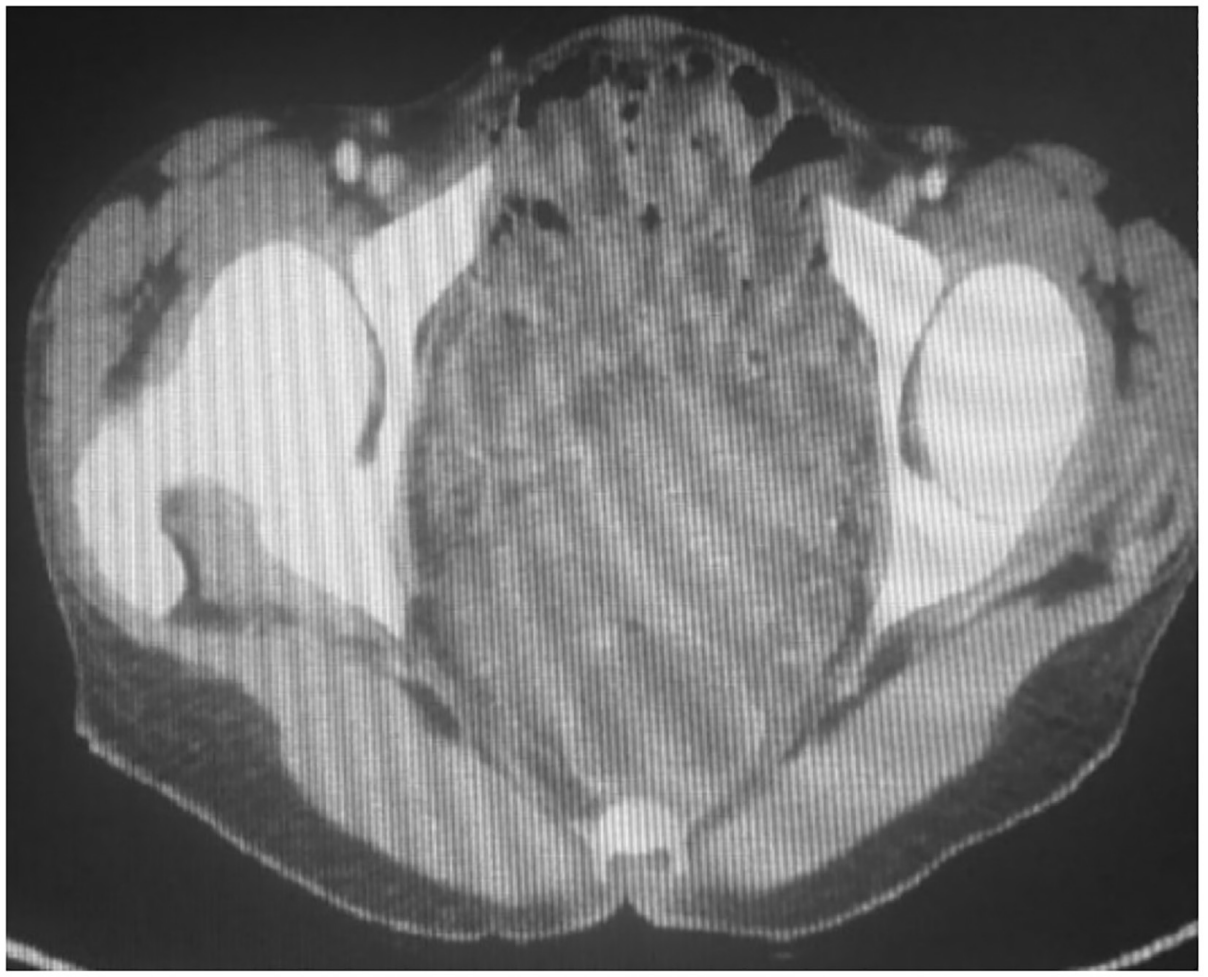
CT scan of the abdomen revealed mildly dilated, fluid‐filled small bowel loops with a fluid plume in the colon surrounded by air bubbles.

**FIGURE 4:**
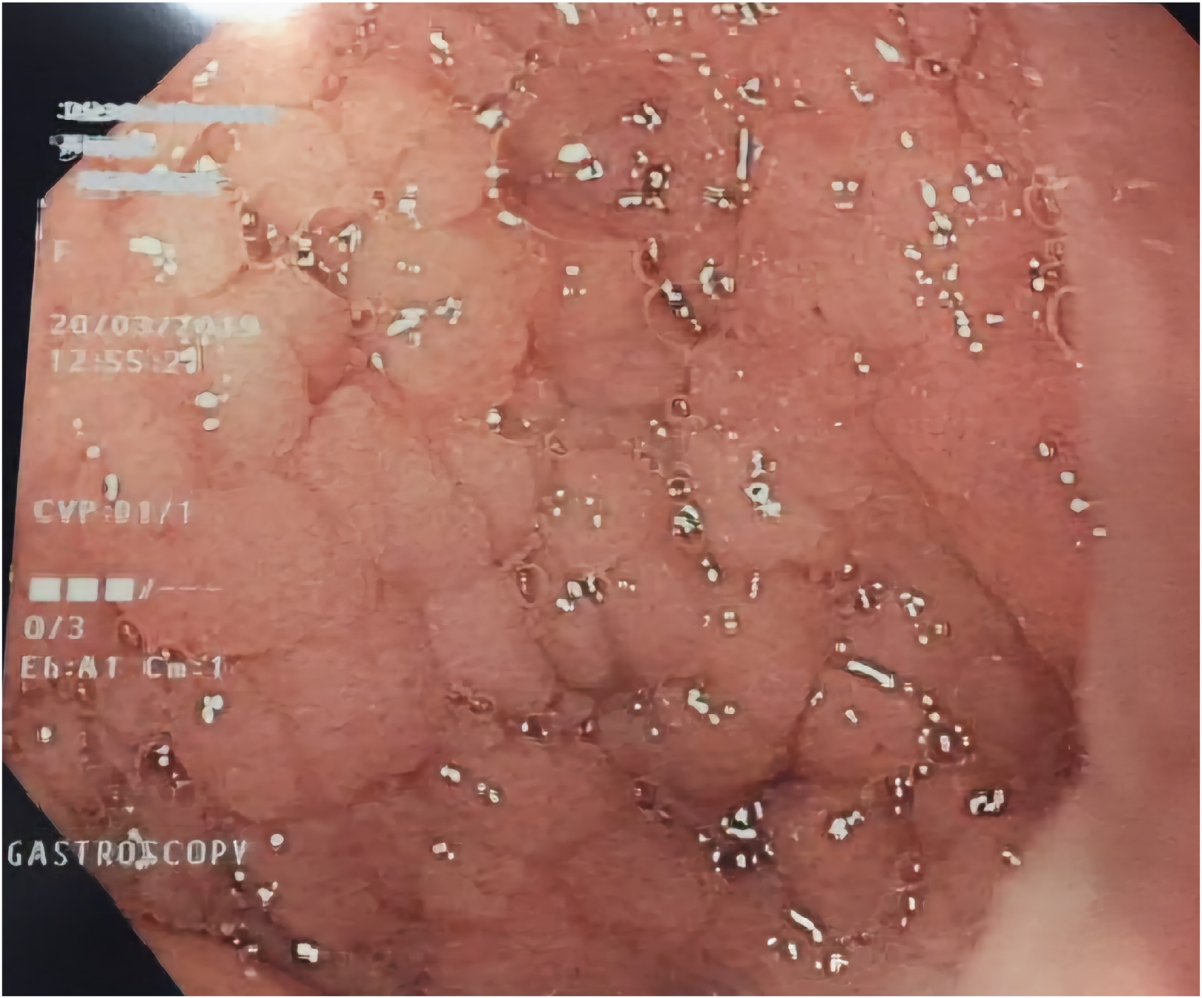
Upper Gastrointestinal endoscopy demonstrating cobblestoning of duodenal mucosa, suggestive of celiac disease.

Given the patient's iron deficiency anemia, low vitamin B12 and folic acid levels, and positive FIT test, a CT scan of the abdomen and pelvis was performed to assess for small bowel pathology. The scan (Figure 3) revealed mildly dilated, fluid‐filled small bowel loops with a fluid plume in the colon surrounded by air bubbles, suggestive of celiac disease. Elevated anti‐transglutaminase IgA levels supported this diagnosis. Endoscopy of the duodenum revealed a cobblestone appearance of the mucosa (Figure 4), and biopsy confirmed severe villous atrophy and crypt hyperplasia, consistent with celiac disease.

## 4 RESULTS (OUTCOME AND FOLLOW‐UP)

The patient was started on a gluten‐free diet, along with regular chest physiotherapy, inhaled salmeterol (50 mcg twice daily), up‐to‐date vaccinations, and azithromycin (250 mg three times per week) for the management of her bronchiectasis. On follow‐up 3 months later, the patient reported significant improvement in both gastrointestinal and pulmonary symptoms, with no new exacerbations. Laboratory tests showed normalization of her iron levels, hemoglobin, vitamin B12, and vitamin D, indicating a positive response to the treatment regimen.

## 5 DISCUSSION

This case highlights the complex interplay between chronic pulmonary disease and gastrointestinal disorders, specifically bronchiectasis and celiac disease. Celiac disease, an autoimmune disorder triggered by the ingestion of gluten in genetically predisposed individuals, leads to chronic inflammation of the small intestine, resulting in malabsorption and a variety of systemic manifestations.^3^ Bronchiectasis, a chronic condition characterized by irreversible dilatation of the bronchi, often results from a cycle of infection and inflammation, leading to airway damage and subsequent impaired mucociliary clearance. Common etiologies include recurrent respiratory infections, immunodeficiencies, autoimmune diseases, and genetic disorders such as cystic fibrosis.^4^

A number of reports have described lung changes in patients with celiac disease including fibrosing alveolitis, sarcoidosis, idiopathic pulmonary hemosiderosis, farmer's lung, asthma, hypersensitivity pneumonitis and lung abscess^5^; however, few investigators reported an association with bronchiectasis. Mahadeva et al. reported a case of bronchiectasis in association with Celiac disease in 1998.^5^ Another case has been described by Ragagopala et al. in 2012 who confirmed the presence of bronchiectasis by high resolution CT scan in a patient who was found to have celiac disease.^6^ To the best of our knowledge no other isolated cases of concomitant bronchiectasis and celiac disease have been reported.

The link between celiac disease and bronchiectasis has been described in the literature, though the exact pathophysiology remains unclear. Some investigators believe that pulmonary changes in patients with celiac disease may be triggered by absorption of an extrinsic allergen or immune complex.^6^ Others support the latter idea linking both celiac disease and bronchiectasis to a common antigen exposure via disturbed gastrointestinal mucosa.^7^ Furthermore, it is believed that a common defect in immunity may lie behind both disorders given the association of celiac disease with HLA status and multiple other autoimmune disease.^8^ It is important to note that both splenic atrophy and IgA deficiency, which are commonly associated with celiac disease, can predispose patients to chronic infections, potentially leading to bronchiectasis. However, our patient did not exhibit either condition and had normal levels of immunoglobulin classes and subclasses. Additionally, no splenic abnormalities were detected on the enhanced abdominal CT scan.^8^

In our patient, the diagnosis of celiac disease was suggested by the presence of iron deficiency anemia, low levels of vitamin B12 and folic acid, and a positive FIT test. These findings prompted further gastrointestinal evaluation, which confirmed the diagnosis of celiac disease. The patient's chronic cough, productive of white sputum, and exertional dyspnea, which had been present for 5 years without medical evaluation, were likely exacerbated by the underlying undiagnosed bronchiectasis. The presence of bronchiectasis in this context raises the possibility that celiac disease may have contributed to the development or exacerbation of her pulmonary condition, possibly through chronic malabsorption and associated chronic inflammation.^2^, ^9^, ^10^ In recent years, there has been growing recognition of the interrelationship between gastrointestinal and pulmonary inflammation. A comprehensive review highlighted the significant prevalence of lung diseases in individuals with inflammatory gastrointestinal conditions, particularly inflammatory bowel disease. This connection underscores the importance of considering the respiratory implications in patients presenting with gastrointestinal symptoms, as inflammation can extend beyond the gut and impact lung health.^11^

## 6 CONCLUSION

This case illustrates the need for a multidisciplinary approach in the evaluation of patients with bronchiectasis, particularly when the etiology is unclear. Early recognition and treatment of underlying conditions such as celiac disease can significantly alter the disease course and improve patient outcomes. Furthermore, it highlights the importance of considering celiac disease in the differential diagnosis of patients presenting with unexplained bronchiectasis, particularly when accompanied by gastrointestinal symptoms or evidence of malabsorption. Early diagnosis and comprehensive management, including both respiratory and gastrointestinal interventions, are key to improving long‐term outcomes in these patients.

## AUTHOR CONTRIBUTIONS

**Paul Ohanian:** Investigation; visualization; writing – original draft. **Joe Khodeir:** Methodology; validation; writing – review and editing. **Marielena Ohanian:** Conceptualization; data curation; supervision; validation; visualization.

## FUNDING INFORMATION

None.

## CONFLICT OF INTEREST STATEMENT

The authors declare no conflicts of interest.

## CONSENT

Written informed consent was obtained from the patient to publish this report in accordance with the journal's patient consent policy.

## Data Availability

The data used to support the findings of this study are included within the article.
